# Photocatalytic bacterial inactivation by TiO_2_-coated surfaces

**DOI:** 10.1186/2191-0855-3-59

**Published:** 2013-10-04

**Authors:** Silvia Bonetta, Sara Bonetta, Francesca Motta, Alberto Strini, Elisabetta Carraro

**Affiliations:** 1 Dipartimento di Scienze ed Innovazione Tecnologica (DiSIT), Università del Piemonte Orientale “A. Avogadro”, Alessandria, Italy; 2 Dipartimento di Scienze della Sanità Pubblica e Pediatriche, Università di Torino, Torino, Italy; 3 ITC-CNR, Construction Technologies Institute – National Research Council of Italy, San Giuliano Milanese, Milano, Italy

**Keywords:** Titanium dioxide, Antibacterial activity, Bacteria, Photocatalytic disinfection, Photocatalysis

## Abstract

The aim of this study was the evaluation of the photoactivated antibacterial activity of titanium dioxide (TiO_2_)-coated surfaces. Bacterial inactivation was evaluated using TiO_2_-coated Petri dishes*.* The experimental conditions optimized with Petri dishes were used to test the antibacterial effect of TiO_2_-coated ceramic tiles. The best antibacterial effect with Petri dishes was observed at 180, 60, 30 and 20 min of exposure for *Escherichia coli*, *Staphylococcus aureus*, *Pseudomonas putida* and *Listeria innocua*, respectively. The ceramic tiles demonstrated a photoactivated bactericidal effect at the same exposure time. In general, no differences were observed between the antibacterial effect obtained with Petri dishes and tiles. However, the photochemical activity of Petri dishes was greater than the activity of the tiles.

Results obtained indicates that the TiO_2_-coated surfaces showed a photoactivated bactericidal effect with all bacteria tested highlighting that the titania could be used in the ceramic and building industry for the production of coated surfaces to be placed in microbiologically sensitive environments, such as the hospital and food industry.

## Introduction

Environmental disinfection plays a crucial role in the prevention of infectious disease (Mitoraj et al. 2007). In recent years, environmental and opportunistic bacteria have been responsible for a large number of disease outbreaks in a variety of settings. Moreover, the increase in microorganism resistance to commonly applied chemotherapeutics and disinfectants constrain the development of new agents for disinfection. The ability to control and/or destroy microorganisms is therefore of enormous importance to many organisations and industries, such as healthcare, food and drink, water treatment and military industries (Gamage and Zhang 2010). The capacity of photocatalytic materials, such as titanium dioxide (TiO_2_), to degrade organic contaminants in the air and water has been studied for more than 20 years (McCullagh et al. 2007). The semiconductor TiO_2_ is non-toxic and is used as an additive in various products, such as cosmetics, foods and pharmaceuticals, as a white pigment. However, this compound is also a photocatalyst and is widely used as a self-cleaning material for surface coating in many applications (Chawengkijwanich and Hayata 2008; Muranyi et al. 2010). TiO_2_ occurs naturally as rutile, brookite and anatase with rutile and anatase being the photocatalytic active forms. Anatase is the most efficient photocatalyst (Blake et al. 1999). The antimicrobial effect of TiO_2_ photocatalytic reaction was reported for the first time by Matsunaga et al. (1985). These authors investigated the effectiveness of the photocatalytic oxidation in water with several microorganisms, including *Lactobacillus acidophilus* (Gram-positive bacteria), *Saccharomyces cerevisiae* (yeast), *Escherichia coli* (Gram-negative bacteria) and *Chlorella vulgaris* (green algae). Since then, studies on photocatalytic killing have been intensively conducted on a wide spectrum of microorganisms, including viruses, fungi and many species of bacteria. However, most of the works utilized acqueous suspensions of TiO_2_ to investigate its antibacterial properties (Evans and Sheel 2007; Foster et al. 2011; Gamage and Zhang 2010). The biocidal action of the TiO_2_ photocatalyst is frequently ascribed to OH^•^ radicals and other reactive oxygen species (ROS) (Cho et al. 2004; Huang et al. 2000). In particular, some studies have demonstrated that the cell membrane is the primary site of reactive photogenerated oxygen species attack, leading to lipid peroxidation (Kiwi and Nadtochenko 2005; Maness et al. 1999; Nadtochenko et al. 2006). The combination of cell membrane damage and further oxidative attack of intracellular components ultimately results in cell death (Rincon et al. 2004). Other studies have suggest that the mode of action is the photooxidation of coenzyme A, leading to the inhibition of cell respiration and thus to cell death (Vohra et al. 2005).

For the biocidal property of the TiO_2_ photocatalyst, surfaces coated with TiO_2_ were proposed as alternative means for disinfection in water, air and food processing (Alrousan et al. 2009; Chorianopoulos et al. 2011; Gamage and Zhang 2010; Guillard et al. 2008; Murany et al. 2010). The use of the TiO_2_ photocatalyst as an antimicrobial component of construction material has also been proposed (Kuhn et al. 2003), but no information about antibacterial effect of this material treated with TiO_2_ is available.

In the ceramic and building industry, the photoinduced bactericidal effect of TiO_2_ can be of special interest. This is particularly true when the ceramic is going to be placed in microbiologically sensitive environments, such as medical facilities and the food industry (Amezaga-Madrid et al. 2002).

Considering that different antibacterial effect of immobilized or suspended titania was observed in some studies (Mitoraj et al. 2007), the aim of this study was the evaluation of the photoactivated antibacterial activity of TiO_2_-coated surfaces. Different bacterial strains which represent common contaminants of food, environment and healthcare facilities, were used as model organisms (*E. coli*, *S. aureus*, *P. putida* and *L. innocua*). Initially bacterial inactivation ability was evaluated using photocatalytic TiO_2_ powder deposited as a thin layer on glass Petri dishes. The experimental conditions optimized with Petri dishes were used for testing ceramic tiles that were chemically coated with TiO_2_. The photocatalytic activity of TiO_2_-coated surfaces was verified by measuring the methylene blue (MB) degradation rate in water under UV-A exposure, and the relationship with antibacterial activity was evaluated.

## Materials and methods

### Bacterial strains and culture methods

*E. coli* cells ATCC 25922 (Oxoid, Cambridge, UK), *S. aureus* (Biogenetics, Pordenone, Italy) and *P. putida* (provided by Dr. Elisa Gamalero, University of Piemonte Orientale, Italy) were precultured in tryptic soy broth (TSB, Applichem, Darmstadt, Germany) at 37, 37 and 28°C, respectively. *L. innocua* ATCC 33090 (Oxoid, Cambridge, UK) was precultured in TSB with yeast extract (0.6%, Applichem, Darmstadt, Germany) at 37°C.

At the exponential growth phase, bacterial cells were diluted with sterile deionised water to the required cell density (10^4^ CFU ml^-1^), as described by Rincon and Pulgarin (2003) and Gogniat et al. (2006). Bacterial concentrations of these dilutions were confirmed by the standard plating method in triplicates on tryptic soy agar (TSA, Applichem, Darmstadt, Germany).

### TiO_2_-coated Petri dishes

A thin layer of photocatalytic TiO_2_ powder P25 (Degussa, Frankfurt, Germany) was deposited on the bottom of glass Petri dishes (diameter 5 cm). After pre-washing with water and ethanol, the Petri dish bottom was covered with TiO_2_ powder suspended in water (1 mg cm^-2^) and dried at room temperature. The Petri dishes were incubated for 1 h at 150°C to fix the TiO_2_ particles. The TiO_2_-coated Petri dishes were used once for each measurement. TiO_2_-untreated Petri dishes were used as blank reference samples. For the antibacterial activity experiments, the Petri dishes were autoclaved (121°C for 15 min), whereas for the MB degradation activity measurements, the Petri dishes were used without any further treatment.

### TiO_2_-coated ceramic tiles

In this study, a series of pre-commercial experimental samples of photocatalytic TiO_2_-coated ceramic tiles (8 × 8 cm) were used. The TiO_2_ coating was chemically prepared by the manufacturer using a proprietary process. Untreated tiles were used as blank reference samples.

In the antibacterial activity experiments, a glass ring was fixed with inert cement to the photoactive surface of each tile (diameter 5 cm). The tile was then placed into a glass Petri dish (diameter 20 cm), and a wet paper was placed between the tile and Petri dish to maintain humidity. The Petri dishes containing the tiles were autoclaved (121°C for 15 min).

For the MB degradation activity measurements, each ceramic tile was protected on all of its non-catalytic surfaces using Teflon ribbon (on lateral sides) and polyethylene film (on the back side).

### Experimental design for the evaluation of antibacterial activity

**Figure 1:**
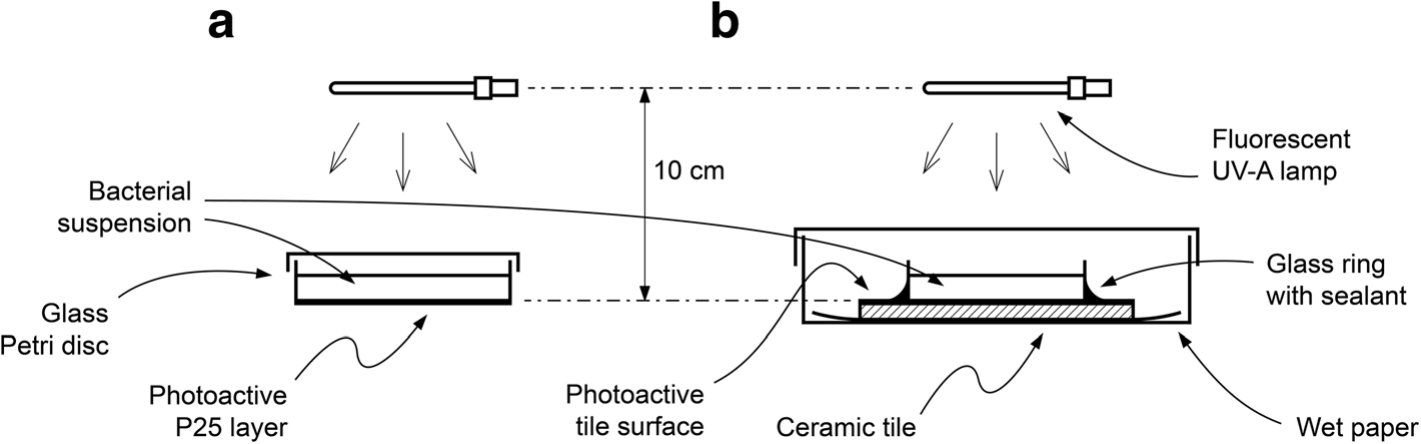
**Schematic illustration of the UV-irradiation system. (a)** Petri-dish system **(b)** Treated tiles system.

The antibacterial activity of TiO_2_-coated surfaces was evaluated in an exposure chamber. In this chamber, the illuminating UV-A light source (wavelength 350–380 nm with irradiance at the sample surface of 9 W m^-2^, Philips) was placed 10.3 cm from the top of the Petri dish, and the temperature during experiments was stabilised using a ventilation system. A schematic illustration of the experimental apparatus is shown in Figure 1(a, b).

An aliquot (100 μl) of a bacterial suspension (10^4^ CFU ml^-1^) resuspended in 400 μl of sterile deionised water was added to a P25-coated Petri dish (TE) and a clean blank Petri dish (BE) to cover the Petri dish surface. Both dishes were exposed to UV-A light. The exposure was carried out for different exposure times: 300 and 180 min for *E. coli* and 180, 60 and 30 min for *S. aureus*, *P. putida* and *L. innocua*. In parallel, a P25-coated (TN) and a clean blank (BN) Petri dishes were inoculated with the bacterial suspension and placed in a dark chamber at room temperature as non-exposed reference samples. During the experiments, the humidity was maintained in all of the Petri dishes with Parafilm sealing.

After the experimental treatment, bacteria were recovered by washing Petri dishes twice with sterile deionised water. The surviving cells were counted by culturing an appropriate dilution of the bacterial suspension in triplicate on agar medium (TSA) for 24–48 h at the appropriate temperature. Each experiment was repeated at least three times. The number of surviving bacteria was expressed as the logarithm of the ratio (survival ratio) of the number of viable bacteria remaining after exposure to experimental conditions (S) to the number of the initial viable bacteria (S_0_) (log S/S_0_).

Analysis of variance (ANOVA, SYSTAT, version 8.0) was used to evaluate the difference between the survival ratio in UV-exposed P25-coated Petri dishes (TE) compared with the other Petri dishes (TN, BE and BN) at different exposure times.

The temperature reached during the experimental trials was measured in a clean Petri dish using a digital thermometer with a K type thermocouple.

An analogous experimental protocol was used for TiO_2_-treated tiles (Figure 1b).

Analysis of variance (ANOVA, SYSTAT, version 8.0) was also used to analyse the differences between the survival ratio in the UV-exposed TiO_2_-treated tiles (TE) compared with the other tiles (TN, BE and BN) at the exposure time selected during the trials conducted with Petri dishes.

### Evaluation of photocatalytic activity

The photocatalytic activity was evaluated by measuring the degradation of MB in water under UV-A exposure. The photocatalytic activity was measured for the P25-coated Petri dishes, TiO_2_-coated ceramic tiles (TE samples) and the uncoated Petri dishes and untreated blank tile samples (BE).

**Figure 2:**
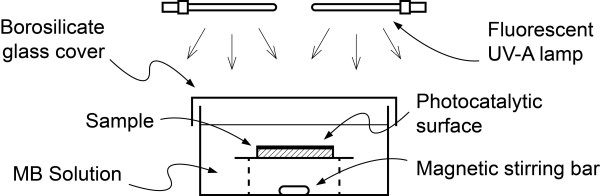
**Experimental set-up for the measurement of the MB photocatalytic degradation activity (not to scale).** The entire system, including the magnetic stirrer (not shown), is enclosed in a closed ventilated box.

All of the measurements were carried out using a batch photocatalytic reactor (Figure 2) with active mixing (provided by a magnetic stirrer). The photocatalytic reactor was closed by a flat glass cover and was operated in a closed exposure chamber equipped with an array of 12 fluorescent UV-A lamps (PL-S/10, Philips). Each sample was placed in the reactor and submersed in 500–700 ml of MB water solution (1.3 ± 0.07 μmol l^-1^), ensuring that the thickness of the solution layer above the catalytic surface was constant (12 ± 1 mm) for all measurements. All of the MB concentration measurements were obtained by determining the absorbance at 662 nm of a 3 mL water solution sample with an UV–vis spectrophotometer (Helios β, Thermo Electron). During each absorbance measurement, the UV lamps were turned off, and after determining the absorbance, the solution sample was re-injected into the reactor vessel. The total measurement time for each sample varied from 6 to 24 h, depending on the photocatalytic activity of the sample.

The irradiance at the sample surface (including the MB solution layer and the reactor cover in the optical path) was measured with a UV-A radiometer (Oriel Goldilux UV-A) and was 500 ± 10 μW cm^-2^ for all experiments. The MB solution temperature during the test was 27 ± 1°C.

The analytical system was calibrated using a standard solution prepared by diluting pure MB (Carlo Erba, Milan, Italy) in deionised water (specific conductance <1 μS cm^-1^). The same MB powder and deionised water were used for the preparation of the working reactor solution.

## Results

### Antibacterial activity of P25-coated Petri dishes

**Figure 3:**
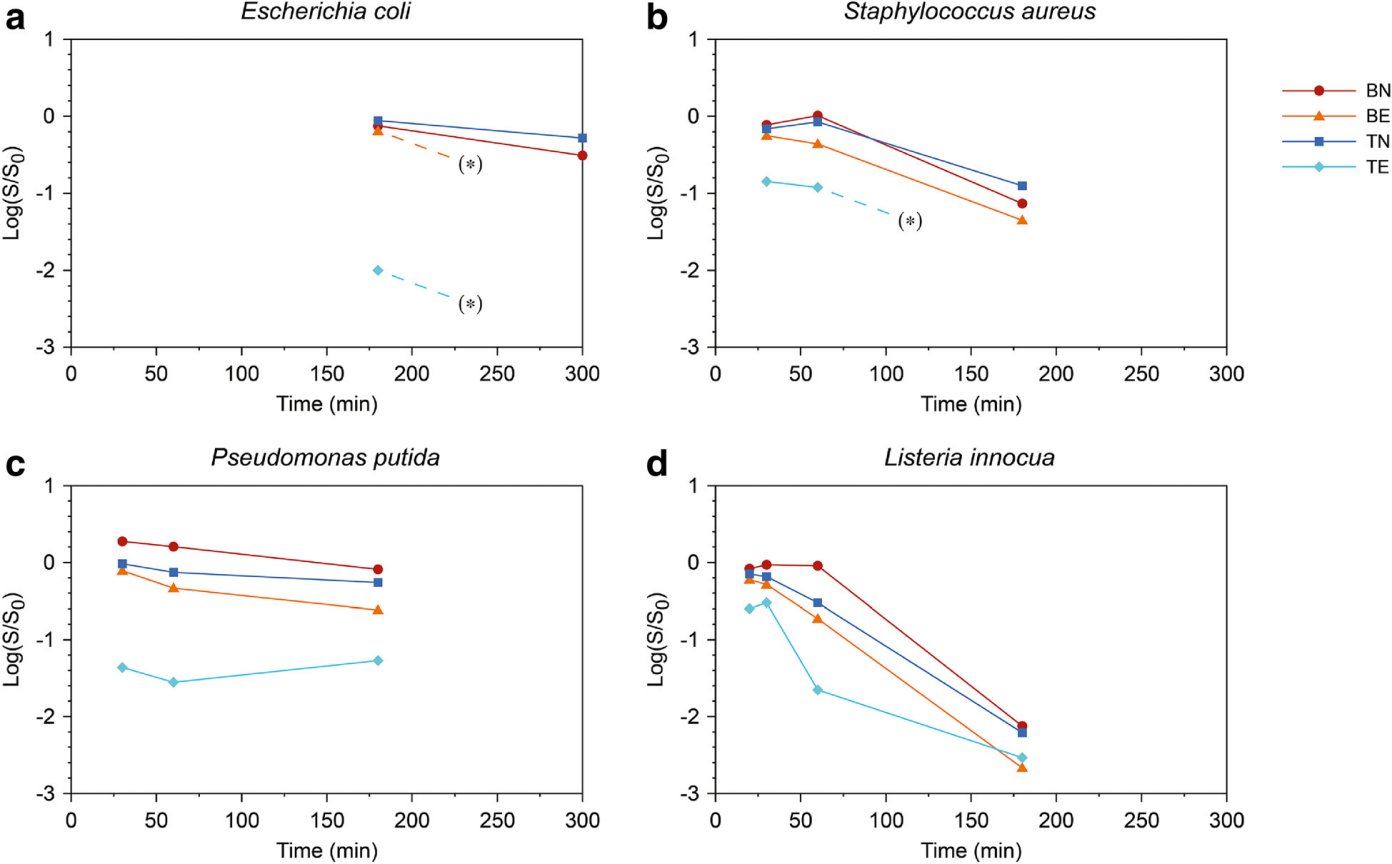
**Antibacterial activity of P25-coated Petri dishes on different bacterial species. (a)***E. coli***(b)***S. aureus***(c)***P. putida***(d)***L. innocua*. Dashed line and (*) indicates an unavailable trend estimation because of the zero survival of the next-step sample.

The results expressed as the logarithm of the surviving bacterial fraction (*E. coli*, *S. aureus*, *P. putida* and *L. innocua*) obtained at different times of exposure are reported in Figure 3. The initial experimental conditions (e.g., exposure time and temperature) were established with the *E. coli* strain because *E. coli* was the first bacterium to be tested during antibacterial trials.

The results obtained with *E. coli* (Figure 3a) showed a low fraction of surviving bacteria in BN and TN samples at 300 min of exposure and no surviving bacteria were observed in any of the exposed Petri dishes (TE and BE). This antibacterial activity could be due to the experimental stress conditions for *E. coli* associated with a long exposure (300 min), such as resuspension in deionised water, light exposure during the sterile operations and UV irradiation.

At 180 min of exposure, good antibacterial activity of TiO_2_ was observed. In fact, the bacterial viability in TE Petri dish was low, whereas a slight reduction of the bacterial level was observed in BE Petri dishes due to the *E. coli* sensitivity to UV radiation. A reduction of bacterial density was also observed in Petri dishes that were not exposed to UV (BN and TN) but at low levels. These results confirm that 180 min of UV exposure is the UV exposure time required to demonstrate the antibacterial activity of UV-activated TiO_2_-coated surfaces with *E. coli*. In fact, at this time of UV exposure, a statistically significant difference between the survival ratio of TE and the other Petri dishes was observed (p < 0.05). Therefore, 180 min was used as the starting exposure time for the trials with the other bacteria.

During the 180 min of exposure, the temperature in a non-TiO_2_-coated Petri dish was monitored to evaluate the effect of this parameter during UV irradiation because in some studies, an increase in temperature was reported during exposure (Vohra et al. 2005). The temperature reached 22.8°C after 30 min of exposure, showing a decrease in temperature compared with the beginning of the experiment (23.9°C). This trend may be related to the ventilation system used in the exposure chamber that allowed air exchange while avoiding temperature increase. After 180 min of the exposure, the temperature was 23.8°C. The results showed that the temperature ranged between 22.8 and 23.8°C with a variation of 1°C, which most likely would not influence the bacterial survival data during the experiment.

The survival curve of *S. aureus* at 180, 60 and 30 min of exposure is reported in Figure 3b. At 180 min of exposure, a reduction of *S. aureus* survival was observed in all of the Petri dishes. The bacterial death observed at this time of exposure could most likely be related to the different stress conditions during the experiment (e.g., resuspension in deionised water and light exposure during the sterile operations), including the long exposure to UV-A radiation. However, the effect of these stress conditions was less evident at 30 and 60 min of exposure, particularly in TN and BN samples. Although low survival was observed in TE dishes at both 30 and 60 min of exposure, the best antibacterial effect in *S. aureus* related to the TiO_2_ treatment was at 60 min of exposure. This finding was confirmed by ANOVA, which showed a statistically significant difference between the antibacterial activity obtained in TE and the other experimental conditions at this time of exposure (p < 0.001).

Regarding the survival of *P. putida* at different times of exposure (180, 60 and 30 min) (Figure 3c), the highest antibacterial effect of TiO_2_ treatment was obtained at 30 min of exposure, at which a statistically significant difference was observed between TE and the other Petri dishes (p < 0.05). Considering the survival data obtained in the BE dishes, a bactericidal effect associated with UV exposure was also observed at 180 and 60 min. Moreover, at 30 and 60 min of exposure, in BN samples a proliferation of *P. putida* was observed. Both of these results highlight a particular resistance of this bacterium under the experimental conditions, most likely due to the environmental origin of Pseudomonadales and of this strain, which was isolated from soil.

The survival curve of *L. innocua* is reported in Figure 3d. As observed for the other bacteria in this study, *L. innocua* appeared to be sensitive to UV starting at 60 min of exposure (BE and TE). Additionally, a reduction of bacterial load was observed for the BN dish at 180 min of exposure, when the experimental stress conditions caused the reduction of living bacteria. Considering the combined UV-TiO_2_ effect, a lower decrease in *L. innocua* load was observed at 30 min of exposure compared with *S. aureus* and *P. putida* (TE dishes). Moreover, at this time of exposure, no statistically significant difference was observed (p > 0.05) between TE and the other experimental conditions; therefore, a further exposure time of 20 min was evaluated for this strain. Under this experimental condition, the difference between TE and the other Petri dishes was statistically significant (p < 0.001).

### Antibacterial activity of TiO_2_-coated ceramic tiles

The described experimental protocol was tested with TiO_2_-coated ceramic tiles to evaluate the antibacterial activity of a real industrial material. The UV exposure times determined by the data obtained in the previous trials with TiO_2_-coated Petri dishes for each bacteria investigated were used during the experiments with the ceramic tiles (180, 60, 30 and 20 min for *E. coli*, *S. aureus*, *P. putida* and *L. innocua*, respectively). In particular, experimental conditions that highlighted the antibacterial effect of TE Petri dishes were selected. These conditions were associated to a statistically significant difference between the antibacterial activity of TE and the other Petri dishes (TN, BN and BE) (p < 0.05 or p < 0.001).

**Figure 4:**
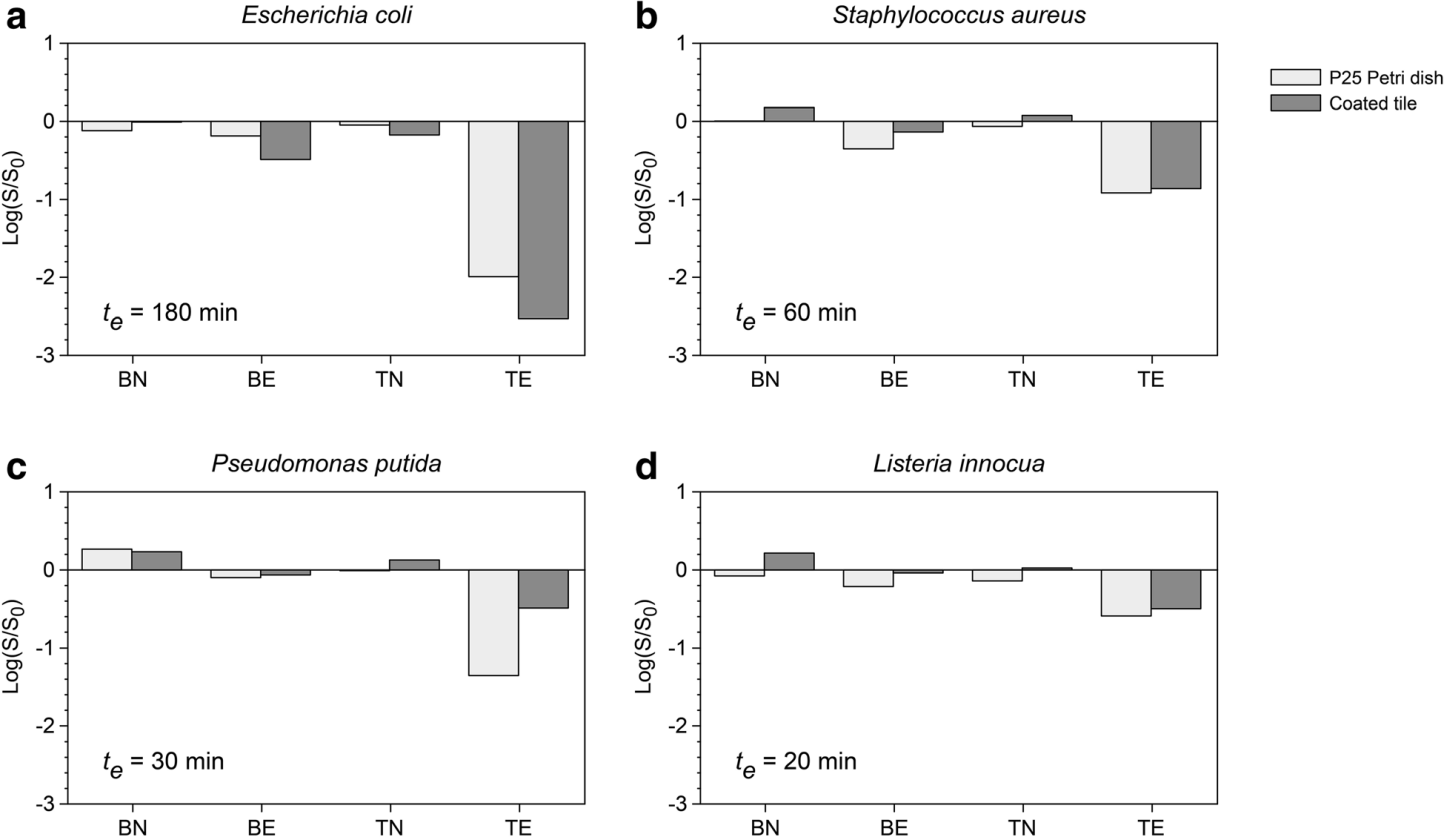
**Antibacterial activity of TiO**_**2**_**-coated ceramic tiles compared with P25 Petri dish on different bacterial species. (a)***E. coli***(b)***S. aureus***(c)***P. putida***(d)***L. innocua*. The exposure time *t*_*e*_ is indicated on each plot.

The survival data of *E. coli*, *S. aureus*, *P. putida* and *L. innocua* are reported in Figure 4 and compared with the results obtained in the Petri dishes at the same UV exposure time. Considering the data obtained with TiO_2_-coated ceramic tiles, a statistically significant reduction (p < 0.05 for *E. coli*, *S. aureus* and *P. putida*; p < 0.001 for *L. innocua*) of bacterial concentration was observed for all of the microorganisms exposed to UV irradiation (TE) compared with non-UV-exposed tiles and controls (BN, BE, TN). Moreover, in general, a slight reduction was revealed in the BE tiles. As observed in the Petri dishes, this trend was most likely related to the antibacterial effect of UV radiation. A low reduction of bacterial survival or proliferation was observed in the BN and TN tiles. Comparing the survival data obtained with ceramic tiles and Petri dishes, no clear difference was observed between the antibacterial effect of the P25-coated Petri dishes versus the treated tiles, except for *P. putida*.

### Evaluation of photocatalytic activity

All of the UV-exposed samples demonstrated first-order degradation kinetics with an exponential decay of the MB concentration, given by the following equation:

(1) $$C\left(t\right)=C\left(0\right)\cdot e^{-\mathrm{kt}},$$

where *C(t)* is the MB concentration at the time *t* (mol m^-3^), *t* is the time (s) and *k* is the exponential decay constant (s^-1^). The constant *k* was calculated for each sample with a non-linear regression of the experimentally measured concentrations and equation (1).

The surface-specific photocatalytic activity of each sample was expressed as the degradation rate *D* at a predetermined reference concentration according to the following:

(2) $$D\left(C_{R}\right)=\frac{C_{R}\cdot k\cdot V}{A},$$

where *D(C*_*R*_*)* is the degradation rate at the concentration *C*_*R*_ (mol m^-2^ s^-1^), *k* is the exponential decay constant, *C*_*R*_ is the reference concentration (mol m^-3^), *V* is the solution volume in the batch reactor (m^3^) and *A* is the photocatalytic sample exposed surface (m^2^).

**Figure 5:**
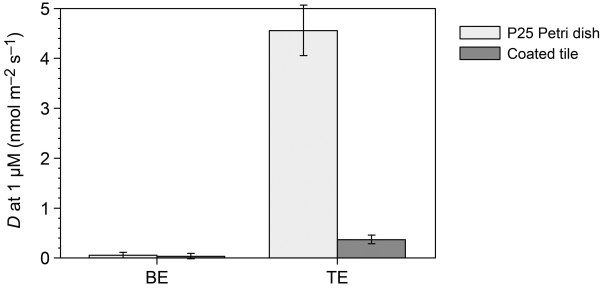
**MB degradation rate at 1 mmol m**^**-3 **^**(1 μM) MB for P25 Petri and tile samples.** The BN and TN samples are not shown because the MB degradation without irradiation is negligible. Estimated errors ± 10 % + 0.05 nmol m^-2^ s^-1^.

The results, expressed as degradation rate at 1 mmol m^-3^ MB (1 μmol l^-1^), are reported in Figure 5. A repeated test on the treated tile demonstrated a ± 5% repeatability error in the resulting *D* value. The degradation of MB in the UV-exposed solution without sample was comparable to the decay measured with the untreated tile sample. The MB degradation of non-exposed TiO_2_-coated and blank samples (TN and BN) was negligible because in the absence of UV, the MB solution is stable, which was also observed in the presence of TiO_2_.

The results showed the higher specific photochemical activity of the P25 Petri sample under these experimental conditions, which was greater than the activity of the treated tile by an order of magnitude. Interestingly, the higher photocatalytic reactivity of P25 samples was not reflected in the antibacterial measurements. In fact, except for *P. putida*, no difference was observed in the antibacterial effect of the P25-coated Petri dishes compared with the treated tiles.

## Discussion

The photocatalytic properties of TiO_2_ are well known and have many applications including the removal of organic contaminants and production of self-cleaning products such as glass, cement and paint (Foster et al. 2011; Strini et al. 2011). There is an increasing interest in the application of the photocatalytic properties of TiO_2_ for disinfection of surfaces, air and water (Gamage and Zhang 2010).

The aim of this study was to evaluate the photoactivated antibacterial activity of TiO_2_-coated surfaces to gain knowledge about a possible application of TiO_2_ as an agent for antimicrobial coating in the ceramic and building industries. At this scope, two different TiO_2_-coated surfaces (P25 glass Petri dishes and ceramic tiles) were tested for antibacterial activity using different bacteria.

The results obtained in the experiment with P25-coated Petri dishes highlighted an antibacterial activity using all microorganisms tested, but demonstrated a remarkable difference in UV-TiO_2_ antibacterial activity for each of the evaluated bacterial strains. There are many hypotheses to explain the different effect of TiO_2_ photocatalysts on various microorganisms. Bacteria may have a self-defence mechanism to protect the cell from oxidative stress (e.g., ROS produced by titania), which may involve enzymes such as catalase and superoxide dismutase (Mitoraj et al. 2007). However, recent studies have highlighted that the cell wall properties (its structure and thickness), rather than the production of antioxidative enzymes seem to have more influence on the survival abilities of bacteria in the presence of titania (Mitoraj et al. 2007). In fact, the susceptibility of the different microbial species may be ordered as follows: gram-negative bacteria > gram-positive bacteria > yeast. Gram-negative bacteria have thin cell walls and gram-positive bacteria have denser cell walls, whereas yeast have a thick eukaryotic cell wall (Foster et al. 2011; McCullagh et al. 2007). However, it is important to note that this susceptibility order was only observed in a limited number of studies (Kuhn et al. 2003). Some investigators have also reported that there is no difference between the TiO_2_ photocatalytic inactivation of gram-positive or gram-negative bacteria. In fact, Tsuang et al. (2008) obtained similar survival ratios in testing different bacteria (*P. aeruginosa*, *S. aureus*, *E. hirae*, *E. coli* and *B. fragilis*) with an orthopaedic implant coated with TiO_2_. Additionally, in a study conducted by Wong et al. (2006), the TiO_2_ sensitivity of some human pathogens (*S. flexneri*, *L. monocytogenes*, *V. parahaemolyticus*, *S. piogenes*, *S. aureus* and *A. baumanii*) was not influenced by whether the target was Gram-positive or Gram-negative bacteria. These results could be related to the use of different experimental systems, such as the type of irradiation (e.g. UV light, solar UV or visible light), TiO_2_ coating/doping with various substances and immobilised or liquid titania. This hypothesis was confirmed by the results obtained by Mitoraj et al. (2007), who observed a different bacterial behaviour of *E. coli* using immobilised or suspended titania.

The results obtained with TiO_2_-coated ceramic tiles showed the photoactivated bactericidal effect of these treated surfaces. Comparing antibacterial effect of the P25-coated Petri dishes versus the treated tiles in general no clear difference was observed. This could probably due to the same contact surface between the bacterial cells and TiO_2_ particles in P25-coated Petri dishes and the treated tiles. These data confirmed the importance of the close contact between the bacterial cells and the TiO_2_ particles in the oxidative damage related to the photocatalytic activity as reported by other authors (Caballero et al. 2009; Foster et al. 2011; Pratap Reddy et al. 2008). Moreover the results obtained with TiO_2_-coated ceramic tiles highlighted that the antibacterial effect of TiO_2_ was present also when this compound was added in a real industrial material.

The photocatalytic dye degradation experiments showed a higher activity in the P25 Petri dishes respect to the tile samples, but these results were not directly comparable with the antibacterial activity. The higher dye degradation observed could be probably due to the ability of MB solution to permeate through the TiO_2_ layer in the treated Petri dishes respect to ceramic tiles. These data demonstrated the requirement for reliable and affordable protocols for the direct measurement of the antibacterial photoactivated activity because the use of an indirect chemical method could provide misleading results.

In summary, results obtained indicates that the TiO_2_-coated surfaces show antibacterial activity with all microorganisms tested highlighting that the titania could be used in the ceramic and building industry for the production of coated surfaces to be placed in microbiologically sensitive environments, such as the hospital and food industry.

## Competing interests

The authors declare that they have no competing interests.
